# Tandem solubility enhancing tags enable the heterologous expression and *in vitro* maturation of a class IIa bacteriocin, clesteriocin a, identified in *Candidatus Clostridium mucoides* CM038

**DOI:** 10.3389/fmicb.2026.1762029

**Published:** 2026-02-13

**Authors:** Joseph Wambui, Taurai Tasara, Laurent Bigler, Roger Stephan

**Affiliations:** 1 Institute for Food Safety and Hygiene, Vetsuisse Faculty, University of Zurich, Zurich, Switzerland; 2 Department of Chemistry, University of Zurich, Zurich, Switzerland

**Keywords:** bacteriocin, *Clostridium estertheticum* complex, genome mining, *Listeria monocytogenes*, pediocin, synthetic biology

## Abstract

The *Clostridium estertheticum* complex (CEC) consists of closely related bacterial species that are mostly isolated from meat processing environment. Genome mining studies have recently established CEC as a source of class I bacteriocins. However, up until now, class II bacteriocins have not been reported from CEC. In the present study, we determined the presence of class II bacteriocin biosynthetic clusters in 33 CEC genomes through genome mining followed by bacteriocin production through heterologous expression in *Escherichia coli*. Six biosynthetic gene clusters belonging to class IIa (*n* = 1), IIb (*n* = 2), IId (*n* = 2) and an undefined class containing three precursor peptides were identified in six different CEC strains. Using molecular biology, we developed dedicated expression vectors for the class IIa bacteriocin, clesteriocin A. Its precursor peptide, CleA, and protease, CleB150, were initially expressed in insoluble form in *E. coli*. Through a systematic analysis of suitable solubility enhancing protein tags, both CleA and CleB150 were expressed in significant amounts of soluble fractions using tandem tags derived from Small Ubiquitin-like Modifier, superfolder Green fluorescent protein, Maltose binding protein and N-utilization substance. Mature clesteriocin A was obtained following *in vitro* maturation reactions between the tagged CleA and CleB150. The novel bacteriocin displayed antimicrobial activity against *Listeria monocytogenes*, which was mediated by the *mptC* gene. By combining genome mining and molecular biology, we have shown CEC is a source of novel class II bacteriocins that have potential for application as food biopreservatives.

## 1 Introduction

Bacteriocins are ribosomally synthesized antimicrobial peptides produced by bacteria known for their potent activity against foodborne pathogens (Bolocan et al., 2017). Unlike chemical preservatives, bacteriocins are generally non-toxic, tasteless, and colorless, making them more appealing to modern consumers’ demands (Deegan et al., 2006). Their potency, heat stability and potential for bioengineering to create derivatives with enhanced properties further enhance their status as promising food biopreservatives. Currently, nisin and pediocin-PA, are the only bacteriocins that have been approved for preservation owing to their generally regarded as safe (GRAS) status and demonstrated effectiveness against a variety of foodborne pathogens (Müller-Auffermann et al., 2015). Depending on their application, bacteriocins can be directly added to foods as purified or semi-purified compounds or produced *in situ* by incorporating the bacteriocin-producing strains as food cultures (Gálvez et al., 2007). These versatile applications, combined with their natural origin and consumer acceptance, position bacteriocins as a sustainable and effective alternative to traditional chemical preservatives in food processing.

Class II bacteriocins, which include pediocin-PA as a notable example, are minimally modified or unmodified thermostable peptides, further divided into four subclasses (Mathur et al., 2015). Class IIa, or pediocin-like bacteriocins, are characterized by the conserved -YGNG(V/L)- motif near the N-terminus and possess one or two disulfide bonds (Todorov et al., 2020). Class IIb require two distinct peptides to function synergistically and form pores in bacterial membranes (Bali et al., 2016). Class IIc are defined by their circular structure, created through the covalent linkage of their N- and C-termini, which further enhances their resistance to heat and proteolytic enzymes (Liu et al., 2023). Class IId are linear, non-pediocin-like peptides lacking conserved motifs, with diverse structures and mechanisms of action (Li et al., 2023).

The mode of action of class II bacteriocins is often potentiated by membrane-bound proteins. For example, many Class IIa, along with some Class IId, bacteriocins use the mannose phosphotransferase system (man-PTS) as a receptor on the target cell membrane (Kjos et al., 2011). This interaction triggers pore formation in the cytoplasmic membrane, resulting in depolarization, inhibition of amino acid uptake, and efflux of pre-imported amino acids. Notably, this efflux occurs independently of the membrane potential, emphasizing the specificity of the man-PTS interaction (Oftedal et al., 2021).

A previous genome mining study identified *Clostridium estertheticum* complex (CEC), which is composed of 11 closely related spore-forming species that are found in meat and meat processing environment (Wambui et al., 2021, 2022a), as a source of class I bacteriocins (Wambui et al., 2022b). It was demonstrated that these bacteriocins have potential for application against foodborne pathogens and clinically relevant pathogens (Guo et al., 2023; Wang et al., 2025). Despite studies showing the potential of CEC as an underexplored yet rich source of novel bacteriocins, until now there are no reports of class II bacteriocins from the complex. Therefore, the current study aimed at leveraging on an expanded collection of sequenced CEC strains to carry out genome mining for class II bacteriocins. Moreover, by applying molecular biology the development of dedicated and efficient expression systems for class II bacteriocins and their cognate peptidases of CEC was established, based on the newly identified bacteriocin, clesteriocin A.

## 2 Materials and methods

### 2.1 General experimental details

Microbial growth media were purchased from Thermo Fisher Scientific, USA, while chemicals and other reagents were sourced from Sigma-Aldrich, USA, unless stated otherwise. The strains and plasmids (Supplementary Table 1) as well as primers (Supplementary Table 2) used in the present study are listed. All strains were previously maintained at −80 °C in appropriate media supplemented with 20% glycerol. Synthetic DNA fragments were designed *in silico* using CLC Main Workbench v22.0.2 (Qiagen, Germany) and synthesized externally by GenScript, Netherlands. Primers synthesis and DNA sequencing services were provided by Microsynth AG, Switzerland.

### 2.2 Isolation of *Clostridium estertheticum* complex strains

Meat juice samples of unspoiled meat (*n* = 1,410) were screened for CEC by quantitative real-time PCR (qPCR) as previously described (Wambui et al., 2022a). PCR positive samples (*n* = 38) with a cycle threshold of <30, which corresponds to approximately 100 spores per ml (Brightwell and Clemens, 2012), were targeted for the isolation of CEC strains. This was carried out anaerobically at 8 °C as previously described (Wambui et al., 2022a). Colonies on Columbia Blood agar supplemented with 5% Sheep blood (CBA) displaying CEC characteristics (Húngaro et al., 2016) were selected and purified twice anaerobically on CBA for 10 days at 8 °C. The isolates were confirmed as members of CEC by qPCR (Wambui et al., 2022a).

### 2.3 DNA extraction and whole genome sequencing

Genomic DNA was extracted using MasterPure Complete DNA and RNA Purification Kit (LGC Biosearch Technologies, United Kingdom). Draft genome sequencing of the strains was carried out as previously described (Wambui et al., 2021). The sequencing outputs (150–300 bp pair-ended reads) were prepared using Nextera DNA Flex chemistry using the Nextera DNA Flex Library Preparation Kit (Illumina, USA) as per manufacturer’s guidelines. The resulting transposon-based libraries were sequenced on a MiniSeq sequencer with a minimal coverage of 50-fold. The MiniSeq MidOutput Reagent Cartridge (300 cycles) was used. Demultiplexing and adapter trimming was done using the Miniseq local run manager v2.4.1 using standard settings. The reads were checked for quality using FastQC (Andrews, 2010) then assembled with SPAdes v3.12.0 (Bankevich et al., 2012) using Shovill v1.0.9.1. The quality of the genomes was checked using ContEST16S (Lee et al., 2017) and CheckM (Parks et al., 2015).

Complete genome sequencing was carried out using MinION Oxford Nanopore Technology (ONT; Oxford Nanopore Technologies, United Kingdom) as previously described (Wambui et al., 2022b) using genomic DNA extracted above. An ONT library was prepared using the 1D ligation sequencing kit (SQK-LSK109) and the native barcoding expansion kit (EXP-NBD104), and sequenced with MinION MK1b device using a R9.4.1 SpotON flow cell. The ONT reads were base-called and demultiplexed using Guppy software (v4.4.1). Trimming and size end filtering were carried out using Cutadapt v2.5. The genome sequences were assembled *de novo* and circularized using Unicycler v0.4.8 run with default parameters, using the paired-end Illumina reads and ONT reads larger than 10 kb (Wick et al., 2017).

### 2.4 In-silico based species delimitation of new CEC strains

We previously established the *rpoB* gene as a reliable marker for phylogenetic classification within the CEC (Wambui et al., 2021). Therefore, the complete *rpoB* sequences of the newly sequenced strains were identified *in silico* and the phylogenetic classification carried out using the in-house method. The correct species assignment was carried out *in silico* through Average Nucleotide Identity (ANI) using EzBioCloud web server (Yoon et al., 2017b) and digital DNA–DNA Hybridization (dDDH) using DMSZ’s Genome-to-Genome Distance Calculator web server (Meier-Kolthoff et al., 2013).

### 2.5 Genome mining for class II bacteriocins

Genome mining was carried out using antiSMASH v6 (Blin et al., 2020). The analysis targeted gene clusters encoding genes associated with class II bacteriocin biosynthesis. These included precursor peptides, peptidase-containing ABC transporters (PCAT), accessory proteins, immunity proteins, transporters and regulators. The search was restricted to clusters whose precursor peptides contained the conserved L(-12)-(X)_3_-E(-8)-L(-7)-(X)_2_-I(-4)-X-G-(G/A) motif (Dirix et al., 2004; Wang et al., 2011; Eslami and van der Donk, 2023), which is recognized by the PCAT for bacteriocin maturation. Based on the precursor peptides and genetic composition, the identified gene clusters were classified into respective classes using an established criteria (Perez et al., 2014). Where needed, the sequences of precursor peptides were compared with known class II bacteriocins.

### 2.6 Creation of dedicated expression vectors

All vectors were created using a PCR-linearized vector backbone obtained from the pET-28a(+) plasmid (Novagen, USA). A synthetic DNA fragment was designed to introduce a modified T7 promoter sequence from pET28a-T7pCONS-TIR-2-sfGFP (Shilling et al., 2020), an N-terminal 8xHis tag sequence and two BsaI sites for Golden Gate Assembly (GGA) flanking a place holder sequence of *lacZ* gene to create pETCECc. Two additional fragments were designed to modify pET28a-T7pCONS-TIR-2-sfGFP and pET-28a(+) into pETCECa and pETCECb, respectively. The latter two fragments retained all transcriptional and translational elements of the original vectors, but introduced two BsaI sites, which flanked a *lacZ* gene, for GGA.

A vector suite for solubility screening was created using six different synthetic DNA fragments. The fragments included one of six solubility enhancing fusion tags; Small Ubiquitin-like Modifier (SUMO), Thioredoxin (TrxA), Glutathione S-transferase (GST), superfolder Green fluorescent protein (sfGFP), Maltose binding protein (MBP) and N-utilization substance (NusA) at the N-terminal, resulting in the creation of pETCECf5, pETCECf6, pETCECf7, pETCECf8, pETCECf9, and pETCECf10, respectively. Additional elements, including RiboJ (Lou et al., 2012), flexible linkers and TEV recognition sequences were introduced into the new vectors. pETCECf8 contained a stop codon of the *sfgfp* gene preventing expression of downstream genes hence it was tested for its ability to enhance solubility alongside the other tags.

Vectors with two tandem solubility enhancing tags were created to optimize solubility of expressed products. Synthetic DNA fragments were designed to introduce N-terminal NusA-SUMO and NusA-sfGFP tags resulting in the creation of pETCECf12 and pETCECf13, respectively. Similarly, fragments introducing C-terminal NusA-sfGFP and SUMO-sfGFP tags were designed and resulted in the creation of pETCECf14 and pETCECf15, respectively. Some elements from the solubility screening vector suite design were introduced to the new vectors. Additional flexible linkers between the tags were introduced for all vectors and a WELQut protease (Thermo Fisher Scientific, USA) recognition sequence for pETCECf13.

The synthesized fragments were amplified by PCR then cloned into the linear backbone of pET-28a(+) using Gibson Assembly® Cloning Kit (New England Biolabs, USA). A 2.5 μL reaction mixture was used to transform Stellar Competent Cells (Takara Inc., Japan) followed by overnight incubation at 37 °C on LB agar plates containing 50 μg/mL kanamycin. Correctly assembled vectors, which were obtained through PCR screening of colonies, were validated through whole plasmid sequencing.

### 2.7 Cloning and heterologous expression

The precursor peptide gene of clesteriocin A, *cleA*, and the cognate peptidase domain of the peptidase-containing ABC transporter, *cleB150*, were PCR-amplified from the genomic DNA of *Clostridium* spp. CM038 with simultaneous introduction of BsaI restriction sites for GGA. For cloning into the expression vectors containing tandem tags, the sequences of *cleA* and *cleB150* were codon optimized using CLC genomics, synthesized externally then amplified by PCR for subsequent cloning. Each gene was cloned into respective vectors using the NEBridge® Golden Gate Assembly Kit (New England Biolabs). Transformation, growth conditions and validation of correctly cloned vectors was carried out as described above.

The expression vectors (100 ng) were transformed into chemically competent *E. coli* BL21 (DE3) or SHuffle® T7 Express strains (New England Biolabs). Individual colonies from PCR-validated transformants were used to inoculate 10 mL overnight cultures in LB containing 50 μg/mL of kanamycin. The overnight cultures were used to inoculate (1:100) 50 mL of LB, also supplemented with kanamycin, followed by incubation at 37 °C with shaking (200 rpm) until an OD_600_ of 0.7–0.8 was reached. Cultures were then cooled on ice for 15 min before addition of IPTG to a final concentration of 0.5 mM. Cultures were incubated at 25 °C and 180 rpm. At specified time intervals (3 h, 6 h, 12 h and 21 h), samples (1 mL) were collected, centrifuged 3 min at 15,000 g and 4 °C. Samples collected at the end of incubation period and from the medium scale expression cultures described below were centrifuged for 10 min at 8,000 g and 4 °C. The supernatants were discarded, and the bacterial cell pellets were stored at −80 °C until further analysis.

### 2.8 Protein expression and solubility tests

To check for protein induction and expression levels, bacterial cell pellets from 1 mL samples were thawed and resuspended in 200 μL PBS (pH 7.4) and analyzed by Tricine-SDS-PAGE or Glycine-SDS-PAGE. Samples were mixed 1:4 with tricine sample buffer for Tricine-SDS-PAGE or 2:1 with 4X Laemli sample buffer for Glycine-SDS-PAGE, heated at 95 °C for 10 min and then loaded into Mini-Protean Tris-Tricine (16.5%) or Mini-Protean TGX gels (4–20%) precast gels (Bio-Rad Laboratories, USA). After running, the gels were fixed for 15 min in fixing solution (40% ethanol, 10% acetic acid), stained (QC Colloidal Coomassie Stain; Bio-Rad Laboratories) overnight, and destained for 2 h in distilled water.

To determine the solubility of the expressed products, bacterial cell pellets from samples collected at the end of incubation were resuspended in 4 mL binding buffer (20 mM NaH_2_PO_4_, 500 mM NaCl, 20 mM imidazole, 10% glycerol, 0.1% Tween 20, pH 7.5) containing 0.25 mg/mL lysozyme for 30 min at 4 °C and sonicated (VibraCell, 15 s ON, 15 s OFF, 50% amplitude) for 5 min. The samples were centrifuged (45 min at15,000 g and 4 °C), and the supernatants were collected as soluble fractions after being filtered with 0.45 μm filters. The cell pellets were further resuspended in 4 mL denaturing buffer (20 mM NaH_2_PO_4_, 500 mM NaCl, 20 mM imidazole, 8 M urea, pH 7.5), mixed in a rotary shaker for 1 h (25 °C, 25 rpm), and then centrifuged as described above. The supernatant was collected as the denatured soluble fraction and cell pellet was resuspended in 200 μL PBS as the denatured insoluble fraction. All fractions were analyzed by SDS-PAGE as described above.

### 2.9 Medium scale protein production and his-tag purification

Medium scale cultures (200 mL) of bacteria cells expressing CleA and CleB150 tagged with tandem solubility enhancing tags were grown as described above. Cell pellets from the centrifuged cultures were resuspended in lysozyme (1 mg/mL) containing binding buffer (5 mL per gram cell pellet), stirred at 4 °C for 1 h, sonicated (10 min), and centrifuged (45 min at 15,000 g and 4 °C). The supernatant was collected, clarified using 0.45 μm filters then mixed with 1 mL of Nickel-nitrilotriacetic acid (Ni-NTA) resin equilibrated with the binding buffer and incubated on a rotary shaker at 4 °C for 3 h or 12 h. The bound resin was loaded onto an open column, washed with five column volumes (CV) of the binding buffer then with five CV washing buffer (20 mM NaH_2_PO_4_, 500 mM NaCl, 40 mM imidazole, 10% glycerol, 0.1% Tween 20, pH 7.5). Elution was carried out with 7 mL elution buffer (20 mM NaH_2_PO_4_, 500 mM NaCl, 400 mM imidazole, 10% glycerol, 0.1% Tween 20, pH 7.5). The eluted fractions were analyzed by Glycine-SDS-PAGE.

### 2.10 *In vitro* maturation of clesteriocin A

Eluted fractions (75 μL) from the Ni-NTA resin were diluted with 25 μL buffer (20 mM NaH_2_PO_4_, 150 mM NaCl, 10% glycerol, 0.1% Tween 20, pH 7.5) then mixed into four reactions (100 μL each) consisting of different combinations of 75 μL tagged CleA and 25 μL tagged CleB150 (Supplementary Table 3), which were supplemented with 2 mM ATP, 2 mM DTT, and 5 mM MgCl_2_. Cleavage of clesteriocin A was carried out at 30 °C for 16 h. Mature CleA was detected by matrix-assisted laser desorption/ionization time-of-flight mass spectrometry (MALDI-TOF-MS) in the linear mode using a Bruker AutoflexSpeed instrument (Bruker, Germany). The samples from the cleavage reaction were first acidified to pH < 4.0 with 10% TFA and 1 μL was then mixed with 1 μL matrix solution (5 mg/mL 𝛼-cyano-4-hydroxycinnamic acid dissolved in H_2_O/MeCN 1:1 + 0.1% TFA). Finally, 1 μL was spotted on a stainless steel target plate and dried at room temperature. Subsequently, was spotted on each sample, dried at room temperature, and then analyzed to check for the expected mass of mature CleA, [M + H]^+^ m/z 4,700.

Samples from reaction 4 consisting of NusA-sfGFP-CleA and CleB150-SUMO-sfGFP (Supplementary Table 3) were used to further validate the *in vitro* maturation of CleA through antimicrobial activity assay. A cleavage reaction without the tagged CleB150 was used as a control. To further confirm that the activity was linked to CleA, after cleavage reaction, the sample was incubated with trypsin (1 mg/mL) for 2 h at 37 °C followed by protease inactivation for 3 min at 90 °C. For the bioactivity assay, overnight cultures of *L. monocytogenes* EGDe wild type (WT), Δ*mptC* gene deletion mutant (Δ*mptC*), and Δ*mptC* complemented with the WT *mptC* gene (Δ*mptC:mptC*) strains were diluted to 10^5^ cfu/mL in BHI. The creation of the (Δ*mptC* and Δ*mptC:mptC*) strains was carried out prior as described in the section below. Aliquots (180 μL) of the diluted cultures were distributed in duplicate into non-tissue culture treated 96 well microtiter plates (Corning Incorporated, USA), then supplemented with 20 μL aliquots of either BHI or samples from the cleavage reactions. Growth was monitored through optical density (OD_600_) measurements for 21 h at 37 °C in Synergy HT OD reader (BioTek, Switzerland). The area under the curve was determined using the R package ‘*opm*’ (Vaas et al., 2013) as previously described (Wambui et al., 2020).

Further optimization of the *in vitro* maturation reaction 4 was carried out by combining different concentrations of ATP, DTT, and MgCl_2_ (Supplementary Table 4). Optimal conditions were determined using antimicrobial activity assay against *L. monocytogenes* WT as described above. All reactions described in this section were carried out in three biological replicates.

### 2.11 *mptC* gene deletion and complementation in *Listeria monocytogenes*

In-frame deletion mutant of *mptC* gene, which encodes the IIC subunit of the man-PTS (Dalet et al., 2001), was created through homologous recombination using the pLR16-pheS* vector (Argov et al., 2017). A deleted copy of the gene that retained its first six and last 10 codons in addition to 500 bp of homology arms was synthesized externally and cloned into the vector using GGA. Correctly assembled vector was validated through whole plasmid sequencing then used for gene deletion in *L. monocytogenes* EGDe as previously described (Argov et al., 2017). The Δ*mptC* mutant was confirmed through PCR amplification and Sanger sequencing. The *mptC* gene was PCR amplified from the WT and cloned into the site-specific integration shuttle vector pIMK2 vector (Monk et al., 2008) for complementation. The gene was cloned using GGA then the correctly assembled vector, which was validated through whole plasmid sequencing, was introduced into the Δ*mptC* mutant as previously described (Monk et al., 2008). Chromosomal integration was also confirmed through PCR and Sanger sequencing using previously described primers (Lauer et al., 2002).

### 2.12 Data analysis

Data analysis was performed using GraphPad Prism version 10.6.1 (GraphPad Software, USA). Differences in the mean percentage decrease in area under the growth curve (PAUC) of *Listeria monocytogenes* EGDe WT were compared with those of the *mptC* mutant and the complemented strain (Δ*mptC*:*mptC*) using Student’s *t*-test to assess inhibitory activity and mode of action of mature clesteriocin A. Student’s *t*-test was also used to compare the mean PAUC of the WT strain in reaction 4 with and without trypsin treatment. Comparisons among different combinations of ATP, DTT, and MgCl₂ were performed using one-way analysis of variance. Post-hoc analysis were carried out using the Dunnet’s multiple comparison test. Statistical significance was defined as *p* ≤ 0.05.

## 3 Results

### 3.1 Expansion of sequenced CEC strains collection

**Figure 1:**
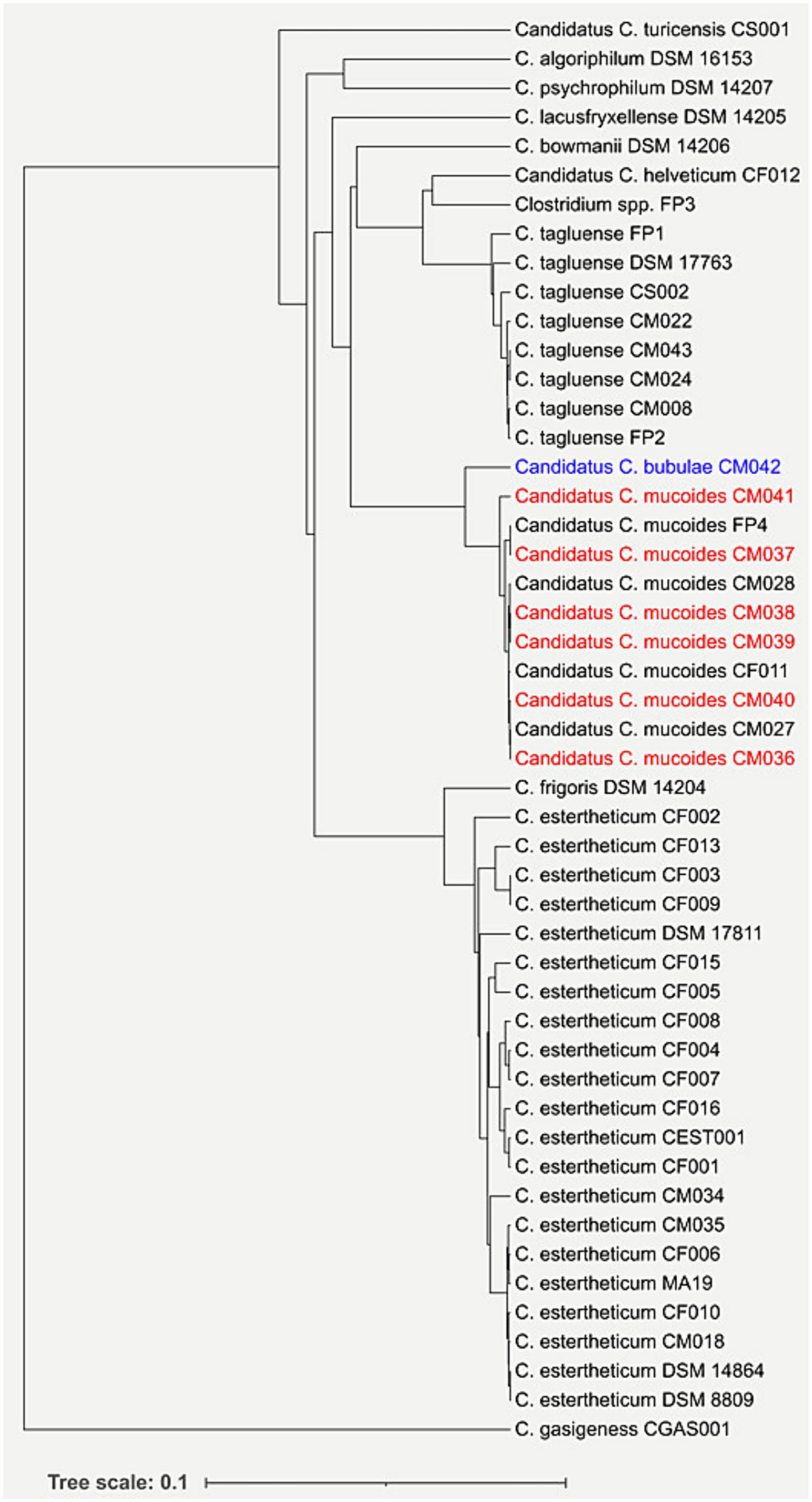
Taxonomic classification of new *Clostridium estertheticum* complex (CEC) isolates based on *rpoB* gene. Newly isolated strains belonging to *Candidatus Clostridium mucoides* are highlighted in red, while a strain belonging to a new species, *Candidatus Clostridium bubulae*, is shown in blue.

Seven strains, which were confirmed by qPCR as members of CEC, were isolated from six meat juice samples. Based on the *rpoB* gene phylogeny (Figure 1), six of the strains (CM036, CM037, CM038, CM039, CM040 and CM041) were identified as members of *Candidatus Clostridium mucoides* (of Mucoides, the Latin name for mucus or slimy, referring to the slimy, mucus-like colonies formed by members of the species (Wambui et al., 2022a)) The species was previously referred to as genomospecies2 (Wambui et al., 2021, 2022a). While strain CM042 although closely related to *Candidatus C. mucoides*, formed its own distinct branch (Figure 1), suggesting it is a novel species. Interspecies dDDH estimates between CM042 and nine members of *Candidatus C. mucoides* ranged from 54.20 to 59.50%, while the ANI values ranged from 93.49 to 94.33%, which were below the 70% dDDH estimate and the 96% ANI value, respectively, for the same species delineation (Table 1). This confirmed that CM042 represents a new species within the CEC, herein named *Candidatus Clostridium bubulae* (of Bubulae, the Latin word for beef, referring to where the strain was isolated). Two other isolates representing new CEC species were also identified in our previous study using the *rpoB* gene phylogeny and phylogenomics and were assigned genomospecies3 and genomospecies4 (Wambui et al., 2022a). To ensure consistency, genomospecies3 and genomospecies4 have been renamed *Candidatus Clostridium helveticum* (from Helvetia, an old name of Switzerland, referring to the country where the respective strain had been isolated) and *Candidatus Clostridium turicensis* (of Turicum, the Latin name for Zurich, referring to the city where the respective strain had been isolated), respectively. The current study has therefore expanded our in-house genome collection to 33, and overall, the genetic diversity of CEC by increasing the number of species to 12.

**Table 1:** Average nucleotide identity (ANI) and digital DNA–DNA hybridization (dDDH) between *Candidatus Clostridium bubulae* CM042 and strains from *Candidatus Clostridium mucoides.*

*Candidatus C. mucoides*	Source	dDDH estimate (%)	ANI value (%)
CF011	Wambui et al. (2021)	55.10	93.61
CM027	Wambui et al. (2021)	54.90	93.61
CM028	Wambui et al. (2021)	55.10	93.53
CM036	This study	55.00	93.65
CM037	This study	59.50	94.33
CM038	This study	56.30	93.94
CM039	This study	55.00	93.55
CM040	This study	55.30	93.56
CM041	This study	54.20	93.49

### 3.2 Evidence of diverse class II bacteriocin biosynthetic gene clusters within CEC

We analyzed the combined 33 CEC genomes for the presence of class II bacteriocin biosynthetic gene clusters leading to the identification of six distinct clusters from six genomes (Supplementary Figure 1). These clusters were broadly classified into pediocin-like bacteriocins (class IIa; *n* = 1), two-peptide bacteriocins (class IIb; *n* = 2), single peptide non-pediocin-like bacteriocins (class IId; *n* = 2) and an undefined class with three peptides (*n* = 1). The precursor peptides of each gene cluster, which contained a conserved L(-12)-(X)_3_-E(-8)-L(-7)-(X)_2_-I(-4)-X-GG motif (Supplementary Figure 2) recognizable by the PCAT for leader peptide removal, are shown in Table 2.

**Table 2:** Characterization of class II bacteriocin biosynthetic gene clusters in *Clostridium estertheticum complex* genomes.

Strain	Species	Class	Precursor peptide sequence^*^
CM038	*Candidatus C. mucoides*	IIa	**MNTLQENELELLNGG**VYYANGVHCDKTHCWVDWGEARGAIGQIVVNGWIQNGPFSHL
CF013	*C. estertheticum*	IIb	**MELNRNFETLETNELENIDGG**SFLLACGIITAGVGVFKLGVEVGKELRSAYDKR **MNNLIELNESDLSEINGG**IWPWLVAVGAAKATPYVVAGTIFVGGVVVGFVNGKK
CS001	*Candidatus C. turicensis*	IIb	**MVELSQNELIDVNGG**GGDDFSHDLGVFVGRKLHDLYDKAMSNSEERNNNKEYWRYKKGVNG **MENINCIELTENELCDVHGG**SVLGGIALFGGVVCAINETYKFGKGVVEGWKNN
CF009	*C. estertheticum*	IId	**MRELRIKEQKSICGG**TMYQFTDLTTGWLYTDTNFYNLYCTRAAASKLHPTHAYSTITP
CM042	*Candidatus C. bubulae*	IId	**MNKLKMSELEQINGG**GPLGGFVVGYLGGKAIDWAVKHPPGKMSPSHKYGQPHGGFL
CF003	*C. estertheticum*	Unclassified	**MDNTFCELNENELMCINGG**GKGDALMTVGGVGVGVGLSCAGEAVTAAFLVSNPIGWGILAGSAAVGAGAYLSSHS **MENIFCELNENELMYINGG**NPGAALIGVGGTAVTAGLGCAGEAVTAAFLVSNPIGWGILAGSAAVGAGVYYINK **MELEYNDGLNELTFTELDEINGG**DGGAFALIGAAMAGGWAFTKAAHETPWVFITVM

* The leader peptide is in bold letters while the GG cleavage site is written in bold.

### 3.3 Description of class IIa bacteriocin gene cluster

**Figure 2:**
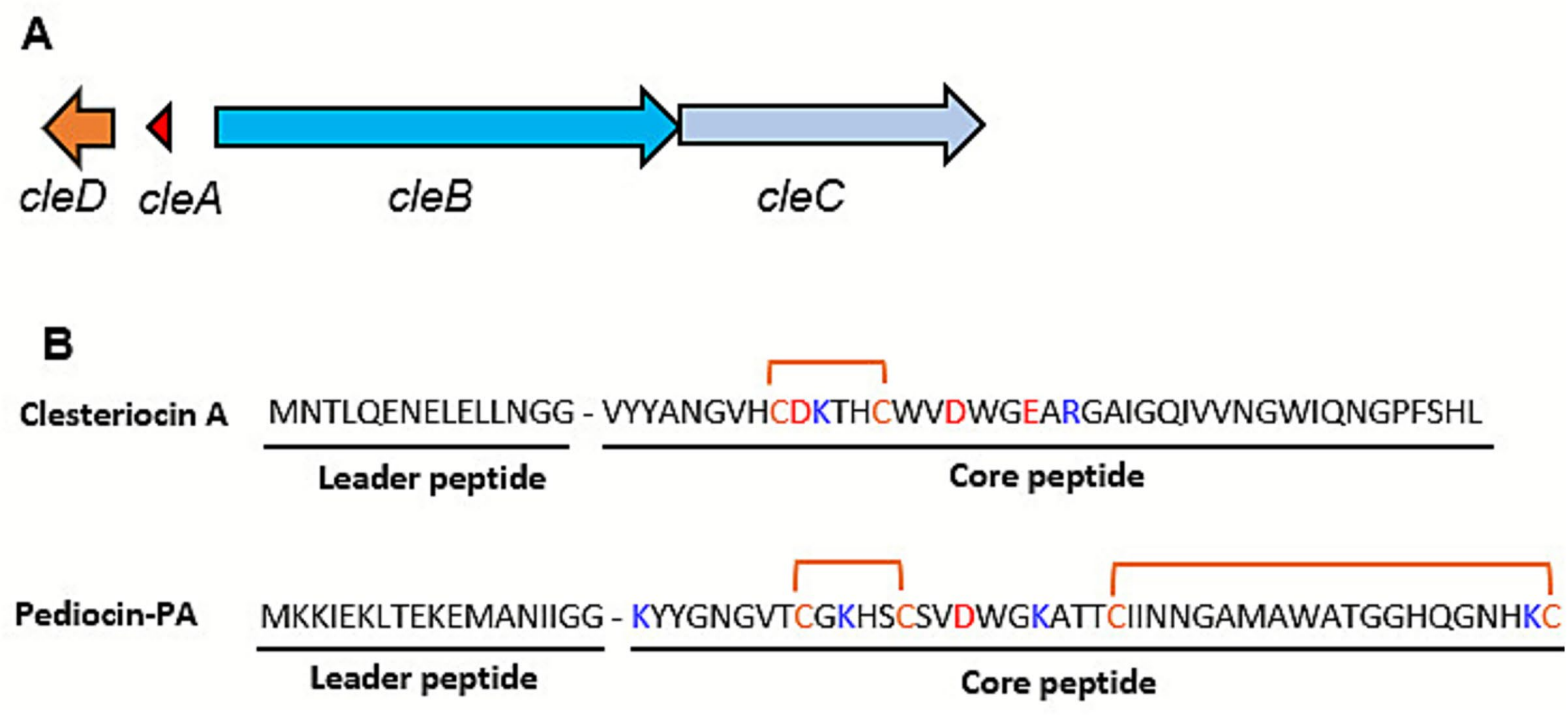
Clesteriocin A is the first class IIa bacteriocin discovered in the *Clostridium estertheticum* complex. **(A)** The gene cluster encodes the precursor peptide, *cleA*, a peptidase containing ABC transporter (*cleB*), an HlyD-type efflux transporter (*cleC*), and immunity gene (*cleD*). **(B)** Amino acid sequence comparison between clesteriocin A and pediocin-PA. Cysteines for disulfide bond formation are shown in orange font. Positively and negatively charged amino acids are shown in blue and red fonts, respectively.

The pediocin-like gene cluster from strain CM038 was selected for heterologous expression in *E. coli* and further characterization (Figure 2). The bacteriocin predicted to be produced by this cluster is here in named clesteriocin A, referring to “ ***Cl*** *ostridium* ***es*** *tertheticum* bact **eriocin** A”. The gene cluster consists of four genes *cleDABC* with *cleA* encoding a 57 amino acid precursor peptide. Upstream of *cleA* is *cleD* encoding the immunity protein while downstream of *cleA*, and transcribed in the opposite direction, are *cleB* and *cleC* for the peptidase containing ABC transporter and a HlyD family efflux transporter, respectively. Compared to pediocin-PA, clesteriocin A differs in the N-terminal pediocin-box by containing a -YANGV- instead of the common -YGNGV- motif. In addition, clesteriocin A has two cysteines compared to four in pediocin-PA and is therefore predicted to form one disulfide bond instead of two bonds that are present in pediocin-PA. Clesteriocin A is predicted to have a net negative charge due to the presence of two positively charged amino acids and three negatively charged amino acids. Pediocin-PA on the other hand has a net positive charge of +3.

### 3.4 Histidine tagged CleA and CleB150 are expressed in *E. coli* in insoluble form

**Figure 3:**
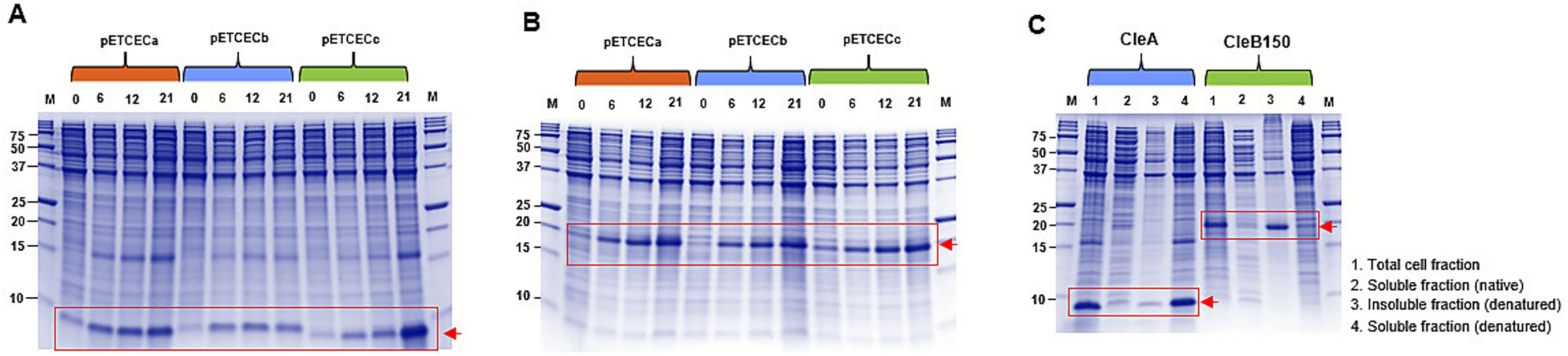
SDS-PAGE analysis of His-tagged CleA and CleB150. Expression levels of **(A)** CleA and **(B)** CleB150 expressed using the developed pETCECc in comparison to pETCECa and pETCECb at various time intervals (0, 6, 12, and 21 h). **(C)** Solubility analysis of expressed CleA and CleB150. Bands representing the expected masses are shown with the red rectangle and arrow.

To achieve heterologous expression of CleA and CleB150 in *E. coli*, a series of in-house pET-based plasmid vectors, pETCECa, pETCECb and pETCECc (Supplementary Figure 3), were developed and used to express the biosynthetic genes, *cleA* and *cleB150*. Expression comparison using the different vectors showed that higher His-tagged CleA expression levels were achieved in pETCECc compared to pETCECa and pETCECb after 21 h of incubation at 25 °C (Figure 3A). Meanwhile His-CleB150 expression levels in the three vectors were comparable at similar conditions (Figure 3B). Based on these observations, CleA and CleB150 expressed by pETCECc were selected for solubility assay. Protein solubility analysis revealed that significant amounts of pETCECc expressed His-CleA and His-CleB150 were however insoluble (Figure 3C). Whereas a significant amount of expressed His-CleA could be solubilized after denaturation with 8 M urea, a significant amount of His-CleB150 remained insoluble (Figure 3C).

**Figure 4:**
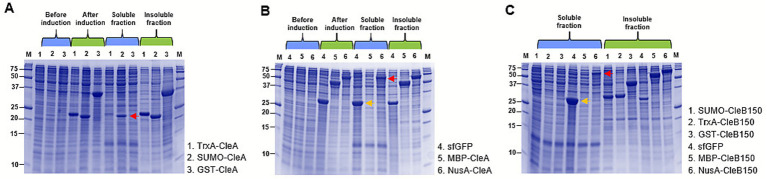
SDS-PAGE analysis of CleA and CleB150 tagged with solubility enhancing tags. Expression levels of CleA expressed with **(A) t**hioredoxin (TrxA), small ubiquitin-like modifier (SUMO), glutathione S-transferase (GST), **(B)** maltose binding protein (MBP), and N-utilization substance (NusA). **(C)** Expression levels of CleB150 expressed with similar tags. The red arrow shows the expected mass of expressed proteins detectable in the soluble fractions. Superfolder GFP (sfGFP) (yellow arrow in the soluble fractions) was tested for its ability to enhance solubility alongside the other tags.

To enhance CleA and CleB150 solubility, a suite of expression vectors incorporating six solubility-enhancing tags was developed. The tags included Small Ubiquitin-like Modifier (SUMO), Thioredoxin (TrxA), Glutathione S-transferase (GST), superfolder Green fluorescent protein (sfGFP), Maltose binding protein (MBP) and N-utilization substance (NusA) (Supplementary Figure 4). Expression of CleA and CleB150 tagged at the N-terminus with these tags revealed their selective role in enhancing solubility. Specifically, SUMO (Figure 4A) and NusA (Figure 4B) improved CleA solubility while only NusA (Figure 4C) improved CleB150 solubility. While these tags resulted in improved solubility, most of the expressed products were still in insoluble form (Figure 4), necessitating further improvements.

### 3.5 Improved CleA and CleB150 soluble fractions using tandem solubility enhancing tags

**Figure 5:**
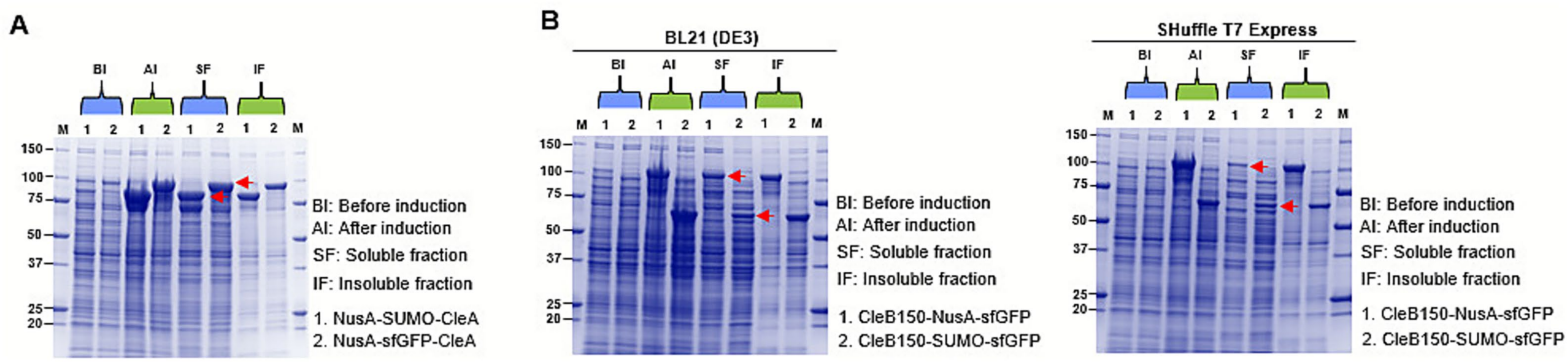
SDS-PAGE analysis of CleA and CleB150 tagged with tandem solubility enhancing tags. **(A)** Expression levels of CleA expressed with N-utilization substance (NusA) and small ubiquitin-like modifier (SUMO) or NusA and Superfolder Gfp (sfGFP) tandem tag. **(B)** Expression levels of CleB150 expressed either in BL21 (DE3) or (SHuffle T7 Express) strains. CleB150 was tagged with a NusA-sfGFP or SUMO-sfGFP tandem tags at the C-terminus. The red arrow show the expected mass of expressed proteins in the soluble fractions.

To solubilize CleA, we created expression vectors pETCECf12 and pETCECf13 that introduced N-terminal NusA-SUMO and NusA-sfGFP tandem tags, respectively (Supplementary Figure 5A). Both tags increased the soluble fraction of tagged CleA when compared to the insoluble fraction (Figure 5A). In case of CleB150, the vectors pETCECf14 and pETCECf15 were created that introduced C-terminal NusA-sfGFP and SUMO-sfGFP tandem tags, respectively (Supplementary Figure 5B). The NusA-sfGFP tag resulted in a higher soluble fraction of CleB150 in *E. coli* BL21 (DE3) strain compared to the SUMO-sfGFP (Figure 5B). Comparing the BL21 (DE3) and SHuffle T7 Express protein expression *E. coli* strains showed the former was most suited for expression of soluble CleB150 fused with the tandem tags (Figure 5B). Therefore, improved solubility of CleA could be achieved after the development of dedicated vectors introducing tandem solubility enhancing tags. In the case of CleB150, introducing the NusA-sfGFP tandem tag in C-terminal resulted in solubility levels comparable to the N-terminal NusA tag, both of which are higher than the expressed non-tagged protease.

**Figure 6:**
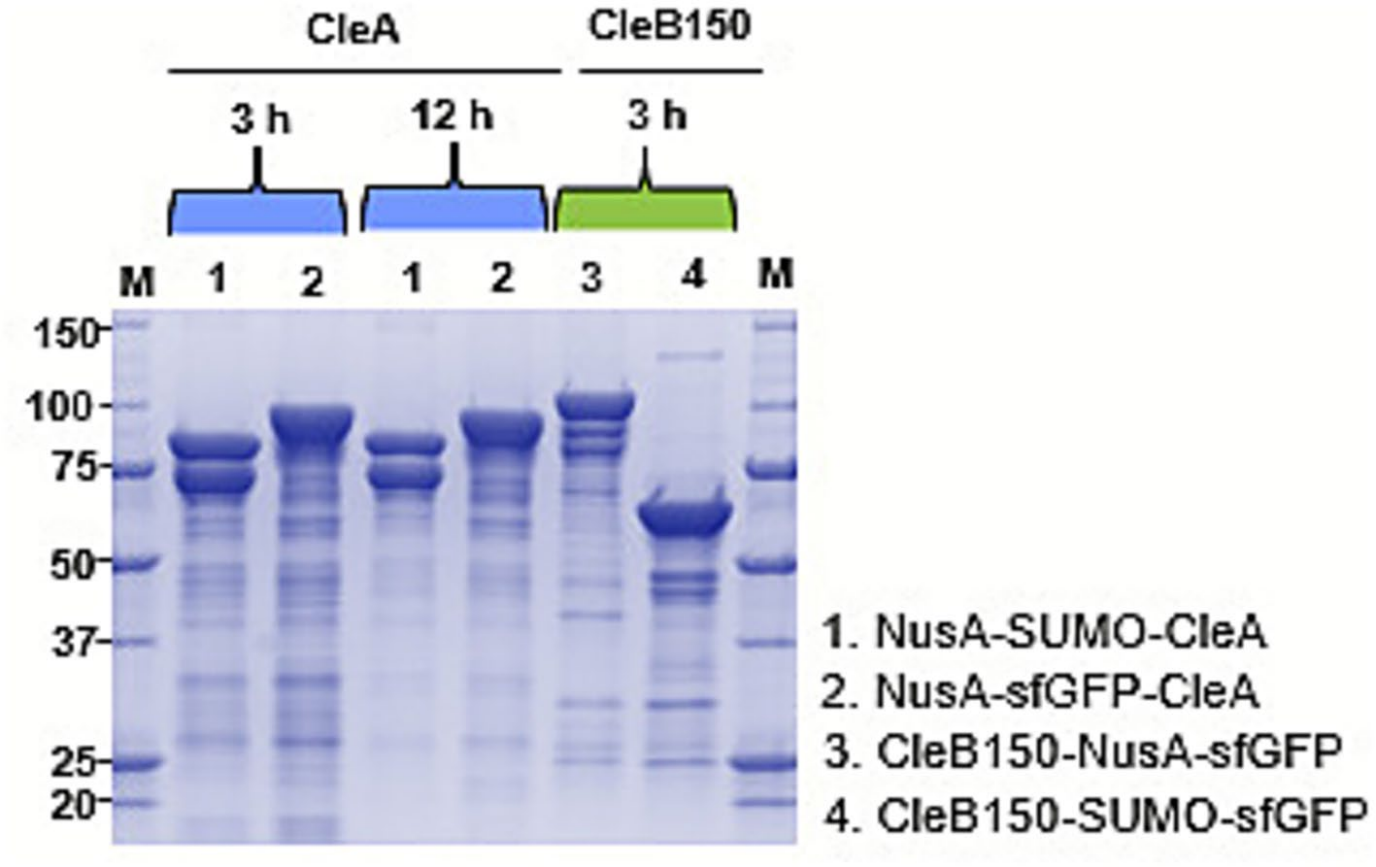
SDS-PAGE analysis of His-purified CleA and CleB150 expressed with tandem solubility enhancing tags. Both CleA and CleB150 were initially incubated at 4 °C with Ni-NTA resin for 3 h for binding, then CleA was incubated again with fresh resin for 12 h.

The 8xHis tag fused at the N-terminus and C-terminus of the tandem tags of CleA and CleB150, respectively, enabled the purification of both products in significant amounts following 3 h incubation with Ni-NTA resin (Figure 6). During His-tag purification, the CleA flowthrough appeared visibly greener due to the sfGFP tag compared to that of CleB150, indicating that the amount of resin used during the initial 3 h incubation was insufficient to bind all CleA. Consequently, fresh resin was added to the flowthrough and the incubation period was extended to 12 h to capture the remaining CleA. Interestingly, this resulted in recovery of more of the precursor peptide. Owing to the observed differences in flowthrough of CleA and CleB150 an additional purification step was not carried for CleB150, as it was theorized that minimal additional protease could be recovered beyond the initial 3 h.

### 3.6 Identification of clesteriocin A

**Figure 7:**
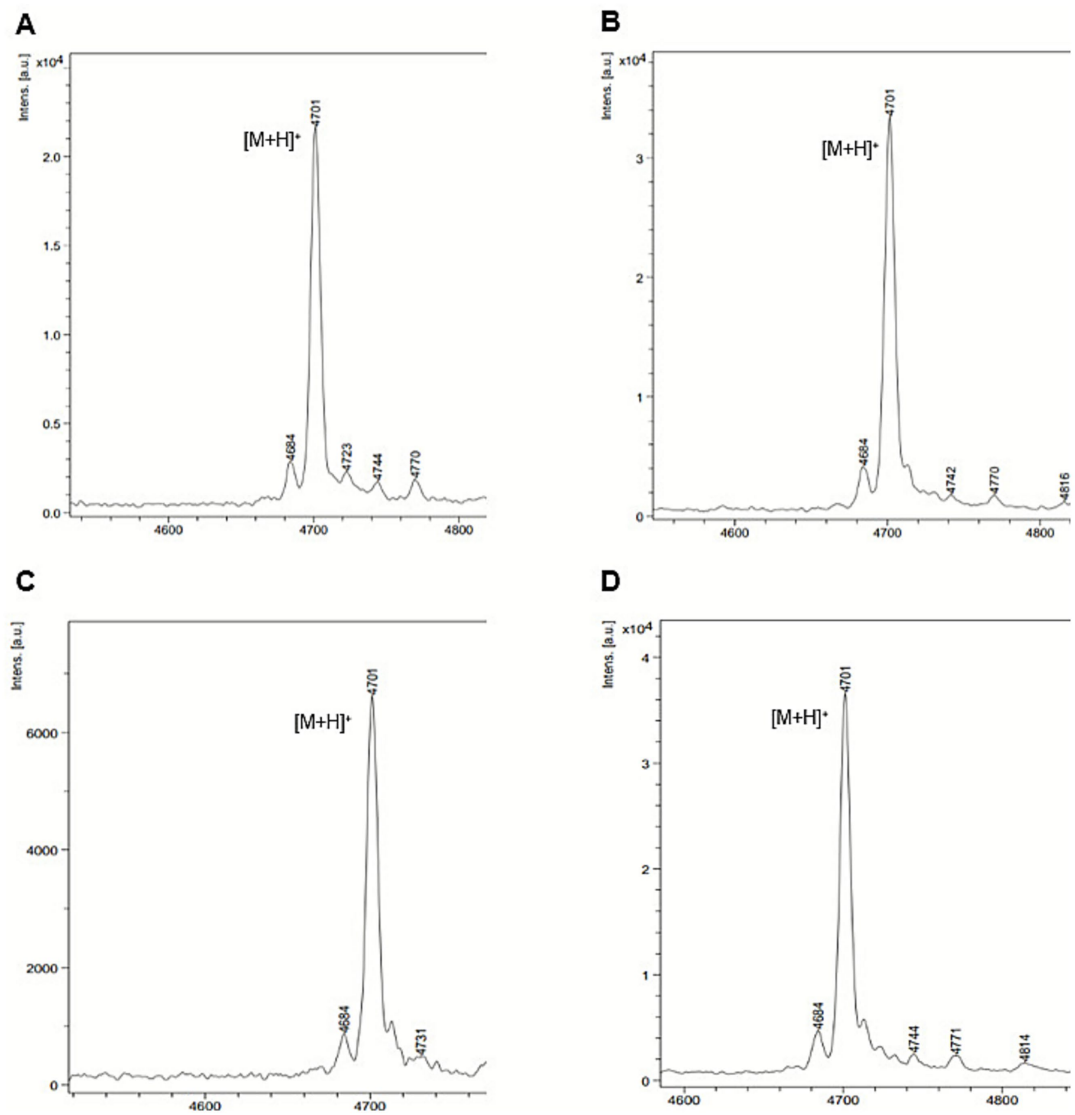
MALDI-TOF MS analysis for mature clesteriocin A after *in vitro* maturation reactions 1–4. **(A)** NusA-SUMO-CleA or **(B)** NusA-sfGFP-CleA reacted with CleB150-NusA-sfGFP. **(C)** NusA-SUMO-CleA or **(D)** NusA-sfGFP-CleA reacted with CleB150-SUMO-sfGFP. The panels **(A–D)** represent reactions 1–4, respectively. The observed mass in all four reactions was [M + H]^+^ *m/z* 4,701 compared to a calculated mass of [M + H]^+^ *m/z* 4,700.

His-purified NusA-SUMO-CleA or NusA-sfGFP-CleA was reacted with His-purified CleB150-NusA-sfGFP or CleB150-SUMO-sfGFP (Supplementary Table 3) to obtain mature clestriocin CleA without the leader peptide. MALDI-TOF analysis, in the linear mode for positive ions, of the four reactions revealed an identical mass of [M + H]^+^ at m/z 4,701, which corresponds to the calculated [M + H]^+^ m/z 4,700 for mature clesteriocin A (Figures 7A–D). Therefore, the tandem tags enabled solubilization of both CleA and CleB150, allowing CleB150 to retain its proteolytic ability thereby enabling the *in vitro* maturation of clesteriocin A. Despite this, it was noted that the cleavage efficiency was variable among the four reactions whereby reaction 4 > reaction 2 > reaction 1 > reaction 3. These variations are most likely as a result of the tag combination in either CleA or CleB or both. It was particularly noteworthy that the reactions with the highest intensity of mature clesteriocin A resulted from reaction 4 and 2 that contained CleB tagged with SUMO-sfGFP. Based on these observations, reaction 4 was selected for subsequent analysis.

### 3.7 Clesteriocin a targets *L. monocytogenes* through the mannose phosphotransferase system

**Figure 8:**
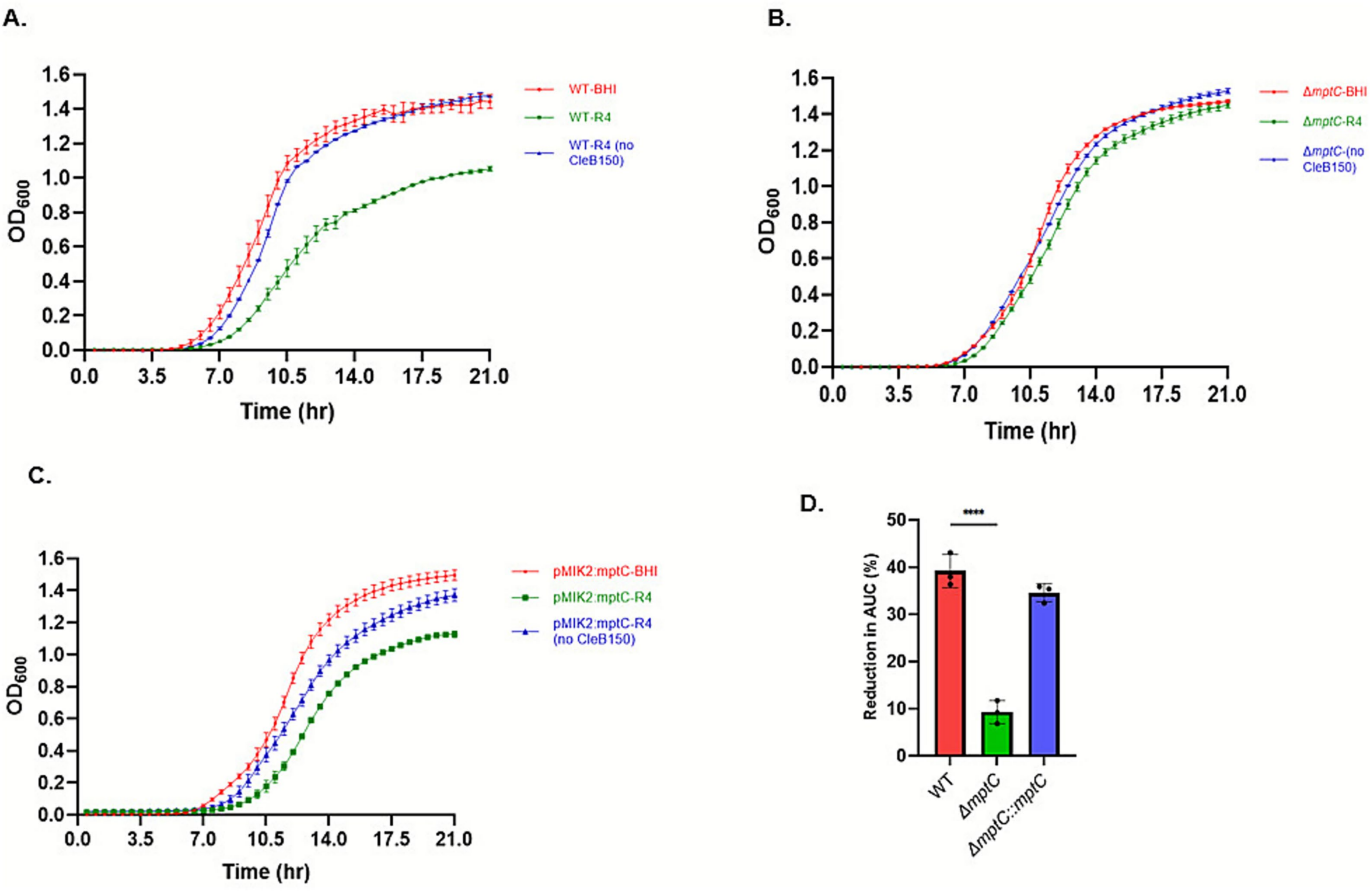
Growth curve analysis of *Listeria monocytogenes* EGDe wild type (WT), *mptC* gene deletion mutant (Δ*mptC*) and *mptC* gene complemented strain (Δ*mptC:mptC*). **(A)** WT, **(B)** Δ*mptC,* and **(C)** Δ*mptC:mptC* strains grown in BHI only or BHI with extract from cleavage reaction R4 (NusA-sfGFP-CleA + CleB150-SUMO-sfGFP) or reaction R4 without CleB150-SUMO-sfGFP. **(D)** Percentage change in the area under the curve (AUC) of WT, Δ*mptC,* and Δ*mptC:mptC* strains grown in BHI only and BHI with the matured clesteriocin A extract from cleavage reaction R4.

**Figure 9:**
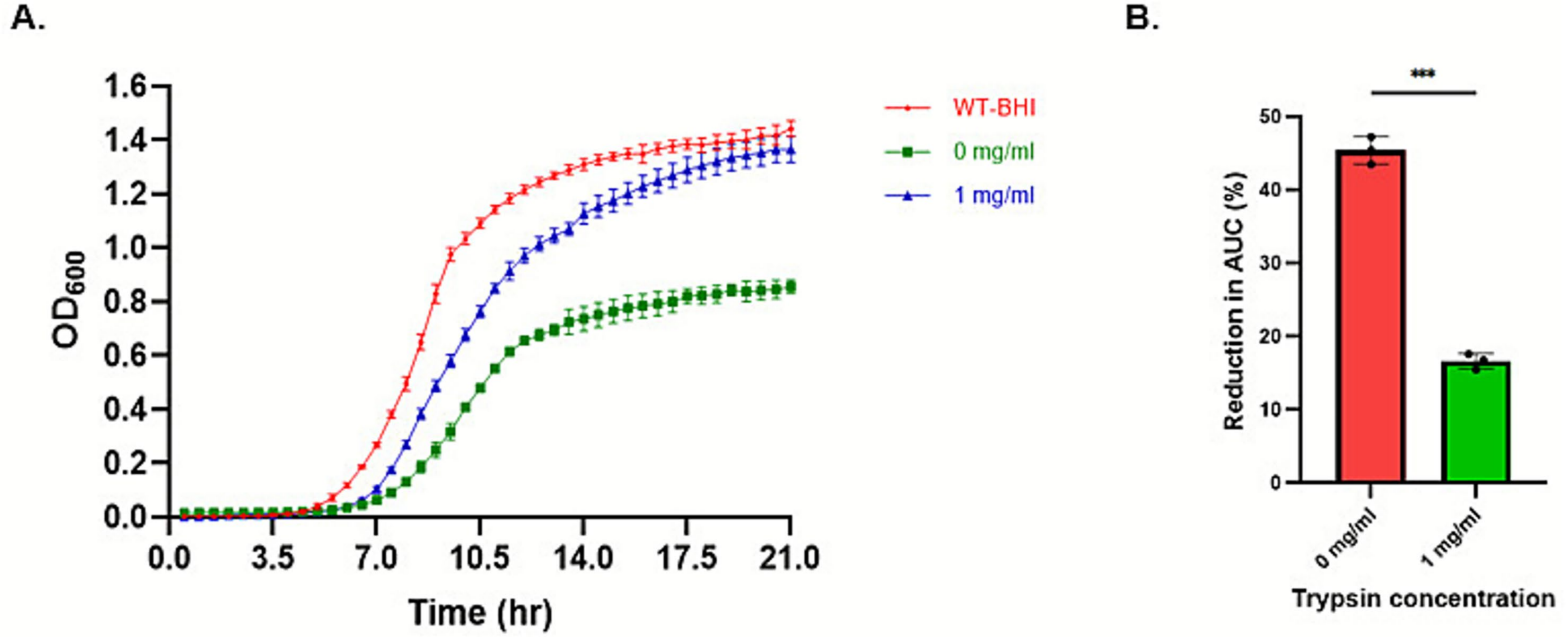
Effect of trypsin on antimicrobial activity of clesteriocin A against *Listeria monocytogenes* EGDe wild type strain. **(A)** Growth curves of the strain in BHI only and BHI with extracts from cleavage reaction4 (mature cleosteriocin A) with or without trypsin digestion. **(B)** Percentage change in the area under the curve of the strain with or without trypsin digestion.

Being a member of pediocin-like bacteriocins, clesteriocin A is predicted to have potent antimicrobial activity against *L. monocytogenes* that is mediated by membrane proteins within the man-PTS. The activity of mature clesteriocin A from the CleB150 processed reaction 4 (Figure 7D; Supplementary Table 3) was therefore tested against *L. monocytogenes* EGDe WT strain, as well as its *mptC* gene deletion mutant *ΔmptC*, and its complemented strain, *ΔmptC*:*mptC*. The *mptC* gene encodes the IIC subunit of the man-PTS, and loss of its function confers pediocin resistance in *L. monocytogenes* (Dalet et al., 2001). Our analysis showed that the growth of the WT strain was significantly impaired upon the addition of the CleB processed mature clesteriocin A preparation from reaction 4 to BHI when compared to the growth of the strain observed in normal BHI (Figure 8A). As expected, there was no growth inhibition of the WT strain observed when BHI was supplemented using a reaction 4 control sample, which had been conducted without CleB150 addition, and thus only contained the unprocessed CleA precursor (Figure 8A). This was in contrast to the *ΔmptC* strain whose growth in BHI was not inhibited upon adding reaction 4 prepared with or without CleB150 (Figure 8B). This indicated that the *ΔmptC* strain was not sensitive to mature clesteriocin A present in the CleB150 processed reaction 4 preparation. Sensitivity of the *ΔmptC* strain to the CleB150 processed clesteriocin A reaction 4 preparation was however, restored through *mptC* gene complementation in the *ΔmptC*:*mptC* strain (Figure 8C). Quantitatively comparing the clestriocin A induced percentage decrease in the area under the growth curve (PAUC) during growth of the three strains in BHI showed the WT strain displayed a significantly large clesteriocin A induced PAUC decrease (*p* < 0.05) compared to the *ΔmptC* mutant but not when compared to the *mptC* complemented mutant strain *ΔmptC*:*mptC* strain (Figure 8D). Combined, the data confirm the antimicrobial activity of the mature clesteriocin A obtained through the *in vitro* maturation reaction. In addition, they confirm that its mode of action also targets the *mptC* gene. Further evidence to support the activity derived from reaction 4 sample is from mature clesteriocin A was obtained through trypsin digestion. Growth of the WT was significantly less impaired upon addition of CleB150 matured clesteriocin A preparation digested with trypsin when compared to the inhibition observed upon addition of the undigested preparation (Figure 9A). In relation to this, the PAUC decrease induced by the trypsin digested clesteriocin A preparation was significantly lower (*p* < 0.05) than that induced by the undigested preparation (Figure 9B). This is consistent with the presence of two positively charged amino acids in clesteriocin A sequence which enable trypsin digestion.

### 3.8 DTT is crucial for the in vitro maturation of clesteriocin A

**Figure 10:**
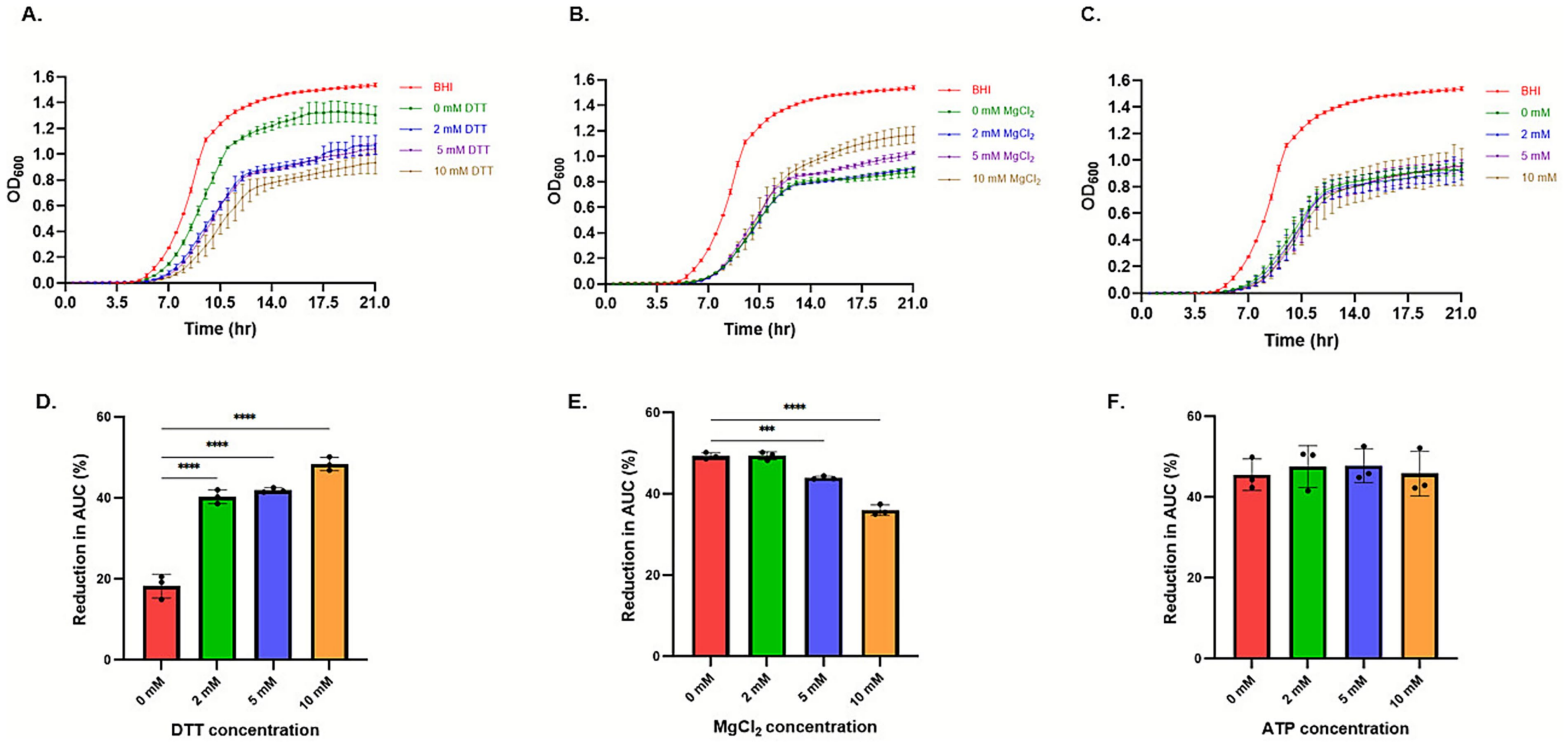
Optimization of clesteriocin A *in vitro* maturation using growth curve analysis of *Listeria monocytogenes* EGDe wild type. Growth curves of the strain in BHI only and BHI with extracts from cleavage reactions supplemented with increasing concentrations of **(A)** DTT, **(B)** MgCl_2_, or **(C)** ATP. Percentage change in the area under the curve of the strain grown in increasing concentrations of **(D)** DTT, **(E)** MgCl_2_, or **(F)** ATP.

We further sought to determine the optimal conditions to obtain the mature clesteriocin A peptide. By testing cleavage reactions supplemented with different concentrations of ATP, DTT and MgCl_2_, it was evident through growth curves and PAUC analysis that DTT and MgCl_2_ concentration had a direct and inverse effect, respectively, on the antimicrobial activity against the *L. monocytogenes* EGDe WT strain, whilst ATP had no significant impact (Figure 10). This was further confirmed by comparing the initial conditions of reaction 4 (2 mM ATP, 2 mM DTT and 5 mM MgCl_2_) against the optimized conditions (10 mM DTT without ATP and MgCl_2_). A slightly, although not statistically significant, more sensitive phenotype of the WT was induced when grown in BHI supplemented with samples of clesteriocin A preparations from optimized conditions compared to initial conditions (Supplementary Figure 6). This indicated that DTT was the most important of the three agents for *in vitro* maturation of clesteriocin A while both ATP and MgCl_2_ were dispensable from the reaction without significantly affecting the mature clesteriocin A activity yield obtained.

## 4 Discussion

Using a targeted genome mining approach, we have for the first time revealed that CEC is a source of class II bacteriocins following the identification of six biosynthetic gene clusters. By narrowing down our search, we identified peptides containing the conserved L(−12)-(X)_3_-E(−8)-L(−7)-(X)_2_-I(−4)-X-GG motif in the leader peptide sequence ensuring a high level of confidence for our genome mining approach. The motif is recognized by the PCAT and plays a key role in bacteriocin maturation and subsequent secretion into the extracellular milieu (Drider et al., 2006). This approach showed that CEC is a source of class IIa, IIb and IId bacteriocins. In addition, we identified a gene cluster that encoded three peptides that fit our genome mining criteria. Whether the three peptides act synergistically as is the case with class IIb bacteriocins warrant future investigations. Our current approach did not permit the identification of circular bacteriocins, which belong to class IIc. Unlike the other classes whose leader peptide is generally cleaved at the double-glycine site, there is no common recognition site for leader cleavage of circular bacteriocins (Zhang et al., 2022). Furthermore, the leader peptides, which often range between 2 and 35 amino acids share no sequence similarity among themselves or those of class IIa, IIb and class IId bacteriocins (Liu et al., 2022).

For heterologous expression in *E. coli*, based on an in-house developed pET-based vector, and for further characterization, the pediocin-like gene cluster from strain CM038 was selected. The pediocin-like bacteriocins are characterized by potent anti-listerial activity and are therefore promising candidates for food biopreservation (Bédard et al., 2018; Khorshidian et al., 2021). Sequence analysis of clesteriocin A revealed a novel pediocin-box -YANGV- motif, which has not been previously reported and differs from the conventional -YGNG(V/L)- motif (Fimland et al., 2005). The potential for application of clesteriocin A as biopreservative and its unique sequence prompted us to characterize it further. Initial efforts to express the precursor peptide, CleA, and its cognate protease, CleB150, derived from the full length PCAT, CleB, with only an N-terminal His-tag resulted in both products being expressed in insoluble form. His-tagged class II bacteriocins are often heterologously expressed in the insoluble fraction (Moon et al., 2006) although the yields of soluble fraction can be improved after fusion with larger, more soluble proteins (Gibbs et al., 2004; Jasniewski et al., 2008). Testing a panel of commonly used solubility enhancing proteins, namely, SUMO, TrxA, GST, MBP and NusA (Costa et al., 2014; Ki and Pack, 2020), showed that the tags selectively improved the solubility of CleA and CleB150. NusA and SUMO showed promise as solubility enhancers for CleA and only NusA showed promising results for CleB150. Consistent with our results, NusA and SUMO are ideal tags for proteins that are difficult to express in *E. coli* (Davis et al., 1999; Butt et al., 2005). Despite of this, most of the expressed products still remained in the insoluble fraction necessitating further improvements.

To enhance the solubility of CleA and CleB150, we used different combinations of NusA, SUMO and sfGFP to develop tandem tags, each consisting of two solubility enhancing proteins. Although both NusA and SUMO have been extensively studied as solubility enhancing tags, data on the application of sfGFP is scarce. A variant of GFP has been used to express class IIa bacteriocins (Vermeulen et al., 2020), but there are no published data demonstrating its use to express the protease domain of PCAT. When the sfGFP was expressed alongside the other tags, we noted that it was expressed mostly in the soluble fraction (Figure 4). This display of high intrinsic solubility in *E. coli* further prompted its use in the development of tandem tags in the present study. For CleA, N-terminal tandem tags, NusA-SUMO and NusA-sfGFP, were added upstream of the leader peptide to allow simultaneous maturation and tag removal by CleB150. For CleB150, C-terminal tandem tags, NusA-sfGFP and SUMO-sfGFP were developed. The protease domain consists of the first 150 amino acids of PCAT hence a C-terminal tag was deemed more suited than an N-terminal tag as it was unlikely to block the catalytic site of the protease. The robustness and versatility of the tags were shown through an increase in overall yield of the soluble fractions of both CleA and CleB150. Furthermore, the tags did not interfere with the 8xHis-tag allowing partial purification of soluble proteins through Ni-NTA affinity chromatography. Ultimately this enabled the subsequent *in vitro* cleavage that resulted in the maturation of clesteriocin A. An experimental approach is therefore presented for the heterologous expression of CleA and CleB150 and *in vitro* maturation of the bacteriocin using the tandem tags.

The mode of action of class IIa bacteriocins involves binding to membrane proteins within the man-PTS system. Using *L. monocytogenes*, we have shown that deletion of *mptC* gene increased the tolerance of the bacteria to a mature clesteriocin A preparation obtained through CleB150 cleavage in reaction 4. The *mptC* gene codes for a membrane protein that is involved in transport within the man-PTS system (Drider et al., 2006). In *L. monocytogenes*, it has been shown that MptC is the target molecule of the class IIa bacteriocins (Ramnath et al., 2000, 2004). The increased tolerance of the *ΔmptC* strain to the mature cleostericin A preparation from the cleavage reaction 4 sample is consistent with the mode of action of class IIa bacteriocins. This was further validated when the sensitive phenotype was restored to the *ΔmptC* mutant through gene complementation in the *ΔmptC*:*mptC* strain. Binding of class IIa bacteriocins to MptC results in increased cell membrane permeability as a result of formation of poration complexes, causing an ionic imbalance and leakage of inorganic phosphate (Ennahar et al., 2000). These disruptions cause the dissipation of proton motive force, which involves the partial or total dissipation of either or both the transmembrane potential (Δψ) and the pH gradient (ΔpH) (Montville and Chen, 1998). While we have shown clesteriocin A action against *L. monocytogenes* is MptC dependent, future studies will determine whether the clesteriocin A MptC binding results in partial or total dissipation of either or both the Δψ and ΔpH. Similarly, we used trypsin digestion to further validate that the antimicrobial activity of reaction 4 sample was attributed to mature clesteriocin A in the preparation. Lysine and arginine at positions 11 and 22, respectively, would subject clesteriocin A to trypsin digestion resulting in loss of antimicrobial activity, which is consistent with our present data. The data also partially enabled us to demonstrate the protease stability of the bacteriocin. Further tests against diverse proteases as well as pH and temperature ranges will be tested on purified clesteriocin A to comprehensively determine its physicochemical properties. This will be enabled by the optimal conditions for heterologous expression, his-tag purification and *in vitro* maturation established in this study.

## 5 Conclusion

The work on clesteriocin A has led to the application of molecular biology in the development of dedicated and efficient expression systems for class II bacteriocins and their cognate peptidases. These systems are anticipated to expedite the comprehensive analysis of clesteriocin A, and the other bacteriocins identified in this study.

## Data Availability

The data presented in this study are publicly available in the NCBI repository (https://www.ncbi.nlm.nih.gov). The accession numbers for *Candidatus Clostridium mucoides* strains CM036, CM037, CM038, CM039, CM040 and CM041 are JBSJEM000000000, JBSJEN000000000, JBSJEO000000000, JBSJEP000000000, JBSJEQ000000000 and JBSJER000000000, respectively. The accession number for *Candidatus Clostridium bubulae* CM042 is JBSJES000000000.
