# Supplementary material for: Tandem solubility enhancing tags enable the heterologous expression and in vitro maturation of a class IIa bacteriocin, clesteriocin a, identified in Candidatus Clostridium mucoides CM038
Source: Front Microbiol. 2026 Feb 13;17:1762029. doi: 10.3389/fmicb.2026.1762029 (PMC12947273; doi:10.3389/fmicb.2026.1762029)
Supplement: Supplementary file 1 [file Supplementary_file_1.docx]

**Supplementary Table 1**. List of strains and plasmids used in this study

| **Strains** | **Characteristics** | **Reference** |
| --- | --- | --- |
| *Clostridium spp.* CM038 | Source of clesteriocin A gene cluster | This study |
| *Escherichia coli* HST08 | Plasmid construction and maintenance | Takara |
| *Escherichia coli* BL21 (DE3) | Protein expression | Invitrogen |
| *Escherichia coli* SHuffle® T7 Express | Protein expression | New England Biolabs |
| *Listeria monocytogenes* EGDe | Indicator strain | Lab collection |
| *L. monocytogenes* EGDe *ΔmptC* | Indicator strain | This study |
| *L. monocytogenes* EGDe *pIMK2:ΔmptC* | Indicator strain | This study |
| *Micrococcus luteus* DSM 1790 | Indicator strain | Lab collection |
| *Bacillus cereus* CH-85 | Indicator strain | Lab collection |
| *Streptococcus agalactiae* | Indicator strain | Lab collection |
| *Staphylococcus aureus* | Indicator strain, methicillin resistant (MRSA) | Lab collection |
| *Enterococcus faecium* | Indicator strain, vancomycin resistant (VRE) | Lab collection |
| **Plasmids** |  |  |
| pET28a-T7pCONS-TIR-2-sfGFP | *Kan^R^*, with modified *T7* promoter and *6XHis* and *sfGfp* cassette | Shilling et al., 2023* |
| pET28a(+) | *Kan^R^*, with conventional *T7* promoter and *6XHis* | Novagen |
| pETCECa | *Kan^R^*, pET28a-T7pCONS TIR-2 sfGFP derivative with modified *T7* promoter *6XHis*, and *lacZ* cassette flanked by BsaI cloning sites | This study |
| pETCECb | *Kan^R^*, pET28a(+) derivative with conventional *T7* promoter *6XHis*, and *lacZ* cassette flanked by BsaI cloning sites | This study |
| pETCECc | *Kan^R^*, with modified *T7* promoter, *8XHis* and *lacZ* cassette flanked by BsaI cloning sites | This study |
| pETCECa_CleA | *Kan^R^*, pETCECa derivate, expressing *His6-cleA* | This study |
| pETCECb_CleA | *Kan^R^*, pETCECb derivate, expressing *His6-cleA* | This study |
| pETCECc_CleA | *Kan^R^*, pETCECc derivate, expressing *His8-cleA* | This study |
| pETCECa_CleB150 | *Kan^R^*, pETCECa derivate, expressing *His6- CleB150* | This study |
| pETCECb_CleB150 | *Kan^R^*, pETCECb derivate, expressing *His6-CleB150* | This study |
| pETCECc_CleB150 | *Kan^R^*, pETCECc derivate, expressing *His8-CleB150* | This study |
| pETCECf5 | *Kan^R^*, with *RiboJ* cassette, modified *T7* promoter, *8XHis*, *SUMO* tag and *lacZ* cassette flanked by *BsaI* cloning sites | This study |
| pETCECf6 | *Kan^R^*, with *RiboJ* cassette, modified *T7* promoter, *8XHis*, *TrxA* tag and *lacZ* cassette flanked by *BsaI* cloning sites | This study |
| pETCECf7 | *Kan^R^*, with *RiboJ* cassette, modified *T7* promoter, *8XHis*, *GST* tag and *lacZ* cassette flanked by *BsaI* cloning sites | This study |
| pETCECf8 | *Kan^R^*, with *RiboJ* cassette, modified *T7* promoter, *8XHis*, *sfGfp* tag* and *lacZ* cassette flanked by *BsaI* cloning sites | This study |
| pETCECf9 | *Kan^R^*, with *RiboJ* cassette, modified *T7* promoter, *8XHis*, *MBP* tag and *lacZ* cassette flanked by *BsaI* cloning sites | This study |
| pETCECf10 | *Kan^R^*, with *RiboJ* cassette, modified *T7* promoter, *8XHis*, *NusA* tag and *lacZ* cassette flanked by *BsaI* cloning sites | This study |
| pETCECf5_CleA | *Kan^R^*, pETCECf5 derivate, expressing *His8-SUMO-cleA* | This study |
| pETCECf6_CleA | *Kan^R^*, pETCECf6 derivate, expressing *His8-TrxA-cleA* | This study |
| pETCECf7_CleA | *Kan^R^*, pETCECf7 derivate, expressing *His8-GST-cleA* | This study |
| pETCECf8_CleA | *Kan^R^*, pETCECf8 derivate, expressing *His8-sfGfp*-cleA* | This study |
| pETCECf9_CleA | *Kan^R^*, pETCECf9 derivate, expressing *His8-MBP-cleA* | This study |
| pETCECf10_CleA | *Kan^R^*, pETCECf10 derivate, expressing *His8-NusA-cleA* | This study |
| pETCECf5_CleB150 | *Kan^R^*, pETCECf5 derivate, expressing *His8-SUMO-cleB150* | This study |
| pETCECf6_CleB150 | *Kan^R^*, pETCECf6 derivate, expressing *His8-TrxA-cleB150* | This study |
| pETCECf7_CleB150 | *Kan^R^*, pETCECf7 derivate, expressing *His8-GST-cleB150* | This study |
| pETCECf8_CleB150 | *Kan^R^*, pETCECf8 derivate, expressing *His8-sfGfp*-cleB150* | This study |
| pETCECf9_CleB150 | *Kan^R^*, pETCECf9 derivate, expressing *His8-MBP-cleB150* | This study |
| pETCECf10_CleB150 | *Kan^R^*, pETCECf10 derivate, expressing *His8-NusA-cleB150* | This study |
| pETCECf12 | *Kan^R^*, with *RiboJ* cassette, modified *T7* promoter, *8XHis*, *NusA* tag, *SUMO* tag and *lacZ* cassette flanked by *BsaI* cloning sites | This study |
| pETCECf13 | *Kan^R^*, with *RiboJ* cassette, modified *T7* promoter, *8XHis*, *NusA* tag, *sfGfp* tag and *lacZ* cassette flanked by *BsaI* cloning sites | This study |
| pETCECf13 | *Kan^R^*, with *RiboJ* cassette, modified *T7* promoter, *lacZ* cassette flanked by *BsaI* cloning sites, *NusA* tag, *sfGfp* tag and *8XHis* | This study |
| pETCECf15 | *Kan^R^*, with *RiboJ* cassette, modified *T7* promoter, *lacZ* cassette flanked by *BsaI* cloning sites, *SUMO* tag, *sfGfp* tag and *8XHis* | This study |
| pETCECf12_CleA | *Kan^R^*, pETCECf12 derivate, expressing *His8-NusA-SUMO-cleA* | This study |
| pETCECf13_CleA | *Kan^R^*, pETCECf13 derivate, expressing *His8-NusA-sfGfp-cleA* | This study |
| pETCECf14_CleA | *Kan^R^*, pETCECf14 derivate, expressing *cleB150-NusA-sfGfp-His8* | This study |
| pETCECf15_CleA | *Kan^R^*, pETCECf15 derivate, expressing *cleB150-SUMO-sfGfp-His8* | This study |

* Shilling, P. J., Mirzadeh, K., Cumming, A. J., Widesheim, M., Köck, Z., and Daley, D. O. (2020). Improved designs for pET expression plasmids increase protein production yield in *Escherichia coli*. *Communications Biology 2020 3:1* 3, 1–8. doi: 10.1038/s42003-020-0939-8

**Supplementary Table 2.** List of primers used in this study

| **Primers** | **Nucleic acid sequences (5' to 3')** |
| --- | --- |
| pET28 fw | CTGAAAGGAGGAACTATATCCG |
| pET28 rv | ATTTCGCGGGATCGAGATC |
| LacZ fw | AGATCTCGATCCCGCGAAATTAATACGACTCACTATAGGG |
| LacZ rv | GATATAGTTCCTCCTTTCAGCAAAAAACCCCTCAAGAC |
| T7 prom fw | TAATACGACTCACTATAGG |
| T7 term rv | CAAAAAACCCCTCAAGACCCGT |
| cleA-pETa fw | GGCTACGGTCTCTCCATATGAACACTTTACAAGAAAAC |
| cleA-pETa rv | GGCTACGGTCTCTGATGTTAAAGATGAGAGAATGGTC |
| cleA-pETc fw | GGCTACGGTCTCCGATGAACACTTTACAAGAAAACGAATTAG |
| cleA-pETc rv | GGCTACGGTCTCGTGCCTTAAAGATGAGAGAATGGTC |
| cleB-pETa fw | GGCTACGGTCTCGCCATATGAATATATTTAAAAAGTATTATTGTGTAAAG |
| cleB-pETa rv | GGCTACGGTCTCAGATGTTAAAGTTCATTTCCTTTTTCAAAC |
| cleB-pETc fw | GGCTACGGTCTCTGATGAATATATTTAAAAAGTATTATTGTGTAAAGC |
| cleB-pETc rv | GGCTACGGTCTCGTGCCTTAAAGTTCATTTCCTTTTTCAAAC |
| cleA-cecf12 fw | GGCTACGGTCTCTCGGTATGAACACCCTGCAGGAAAACG |
| cleA-cecf12 rv | GGCTACGGTCTCTTGCCTTACAGATGGCTAAACGGGCCG |
| cleA-cecf13 fw | GGCTACGGTCTCTGCAGATGAACACCCTGCAGGAAAACG |
| cleB-cecf15 fw | GGCTACGGTCTCTACATATGAACATTTTTAAAAAATATTATTGCG |
| cleB-cecf15 rv | GGCTACGGTCTCTTACCTTTCTGCGGTTTAATCAGATC |

**Supplementary Table 3.** *In vitro* cleavage reactions between CleA and CleB150 fused with tandem tags

| Tagged CleA | Tagged CleB150 | | | |
| --- | --- | --- | --- | --- |
|  | Reaction 1 | Reaction 2 | Reaction 3 | Reaction 4 |
| NusA-SUMO-CleA | CleB150-NusA-sfGfp |  |  |  |
| NusA-sfGfp-CleA |  | CleB150-SUMO-sfGfp |  |  |
| NusA-SUMO-CleA |  |  | CleB150-NusA-sfGfp |  |
| NusA-sfGfp-CleA |  |  |  | CleB150-SUMO-sfGfp |

**Supplementary Table 4.** Combinations of different concentrations of MgCl_2_, DTT and

ATP for in vitro cleavage reactions

|  | MgCl_2_ (mM) | DTT (mM) | ATP (mM) |
| --- | --- | --- | --- |
| Combination 1 | 0 | 2 | 2 |
| Combination 2 | 2 | 2 | 2 |
| Combination 3 | 5 | 2 | 2 |
| Combination 4 | 10 | 2 | 2 |
| Combination 5 | 5 | 0 | 2 |
| Combination 6 | 5 | 2 | 2 |
| Combination 7 | 5 | 5 | 2 |
| Combination 8 | 5 | 10 | 2 |
| Combination 9 | 5 | 2 | 0 |
| Combination 10 | 5 | 2 | 2 |
| Combination 11 | 5 | 2 | 5 |
| Combination 12 | 5 | 2 | 10 |
| Optimal condition | 0 | 10 | 2 |

**
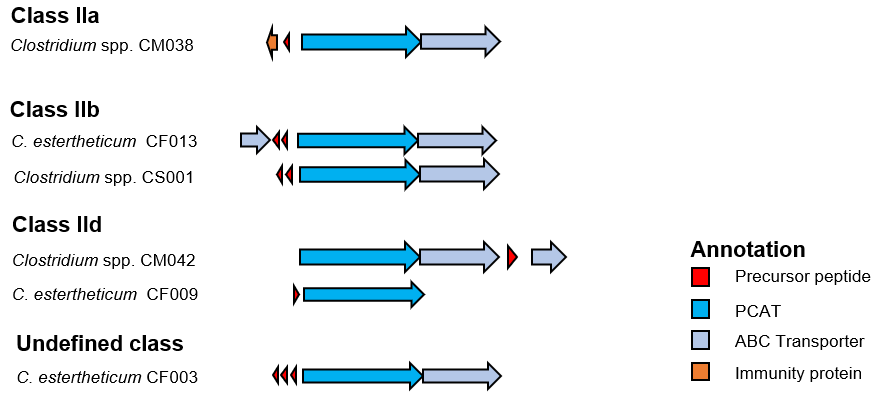
**

**Supplementary Figure 1**. Class II bacteriocin biosynthetic gene clusters identified within *Clostridium* *estertheticum* complex. Each cluster was characterized by the presence of a minimal set of genes required for biosynthesis, mainly a precursor peptide (n = 1 to 3) and a peptidase containing ABC transporter (PCAT) for maturation through leader peptide removal. The gene clusters were not drawn to scale.


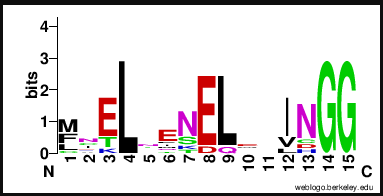


**Supplementary Figure 2**. Consensus sequence logo of identified class II bacteriocin leader peptides.


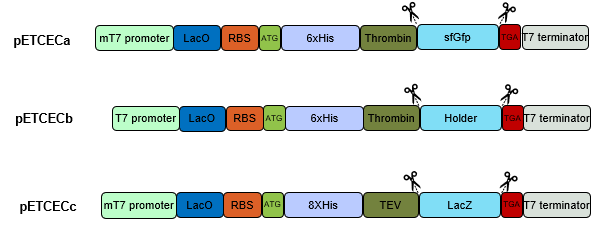


**Supplementary Figure 3**. Development of pETCECc for heterologous expression in *Escherichia* *coli*. The vector is a derivative of both pET28a(+) and pET28a-TIR-2+T7pCONS-sfGFP, which were presently converted to pETCECa and pETCECb, respectively, for Goldengate assembly. pETCECc has a modified T7 promoter (mT7 promoter) for high protein expression, 8xHis tag for affinity chromatography, a TEV recognition site for tag removal and *lacZ* gene sequence as drop out sequence that can also be used for blue-white screening. The scissors symbol represent the BsaI restriction enzyme recognition site for Goldengate assembly.


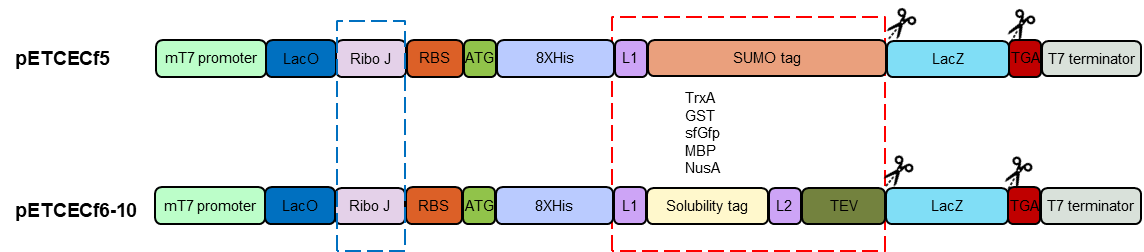


**Supplementary Figure 4**. Development of a vector suite for solubility screening. Modifications were made to introduce N-terminal solubility enhancing tags (red rectangle), which included Small Ubiquitin-like Modifier (SUMO; pETCECf5), Thioredoxin (TrxA; pETCECf6), Glutathione S-transferase (GST; pETCECf7), superfolder Green fluorescent protein (sfGfp; pETCECf8), Maltose binding protein (MBP; pETCECf9) and N-utilization substance (NusA; pETCECf10) upstream of the BsaI cloning site (scissors symbols). A sequence for the self-cleaving ribozyme *ribo* J (blue rectangle) was introduced between the *lacO* operator and ribosomal binding site (*RBS*) sequences to insulate the developed genetic circuits.


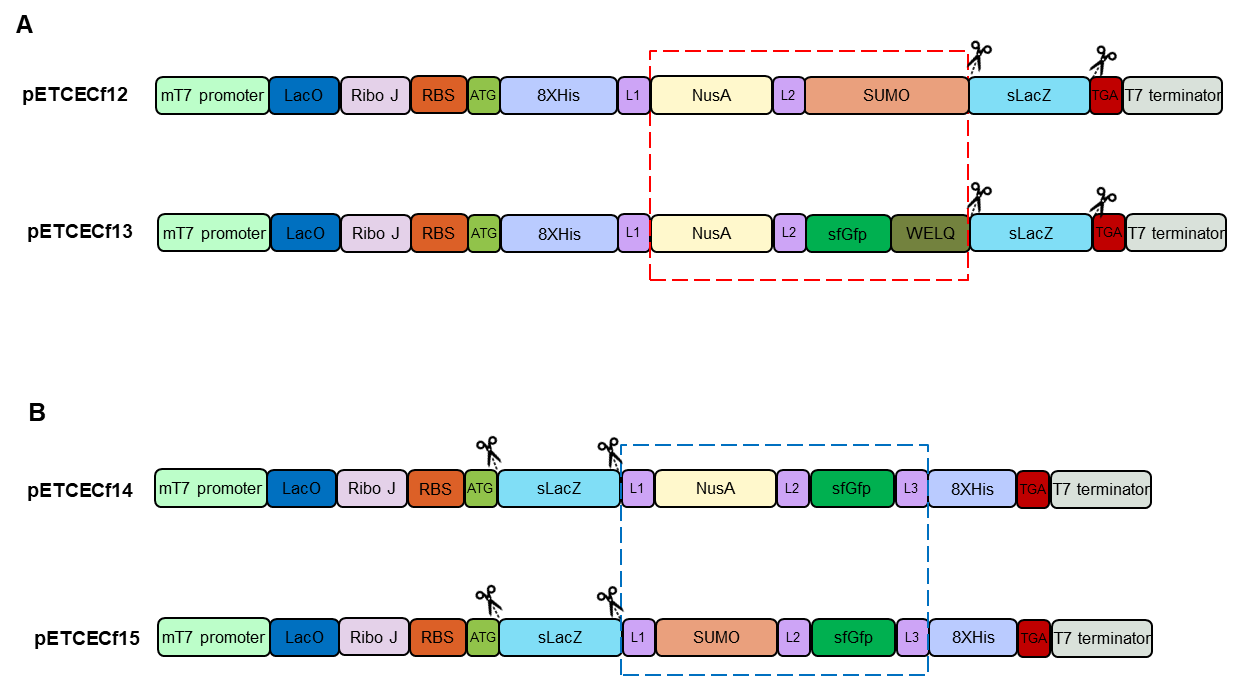


**Supplementary Figure 5**. Development of vectors with tandem solubility enhancing tags. **(A)** Introduction of N-terminal tandem tags (red rectangle) for CleA. N-utilization substance (NusA) and Small Ubiquitin-like Modifier (SUMO) were used to create pETCECf12. NusA was linked with superfolder Green fluorescent protein (sfGfp) to create pETCECf13. **(B)** Introduction of C-terminal tandem tags (blue rectangle) for CleB150. Linking NusA and sfGfp created pETCECf14 while linking SUMO and sfGfp created pETCECf15. The scissor symbols represent the BsaI cloning site flanking a short drop off sequence (sLacZ) of *lacZ* gene.


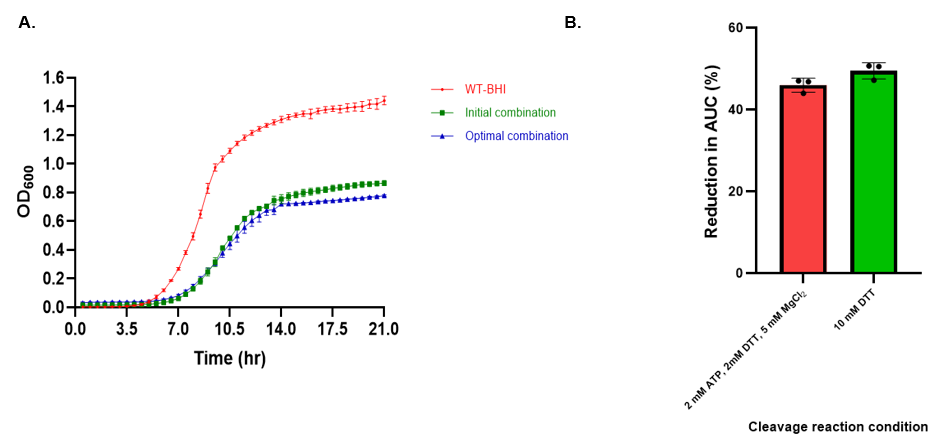


**Supplementary Figure 6**. A comparison between two different combinations of DTT, MgCl_2_ and ATP on maturation of clesteriocin A. **(A)** Growth curves of the strain in BHI only and initial combination of 2 mM DTT, 5 mM MgCl_2_ and 2 mM ATP or optimized combination comprising only 10 mM DTT. **(B)** Percentage change in the area under the curve of the strain grown in the initial combination or optimized combination of additives.
